# Does conserved domain SOD1 mutation has any role in ALS severity and therapeutic outcome?

**DOI:** 10.1186/s12868-020-00591-3

**Published:** 2020-10-09

**Authors:** Surinder Pal, Abha Tiwari, Kaushal Sharma, Suresh Kumar Sharma

**Affiliations:** 1 Centre for Systems Biology and Bioinformatics, Panjab University, Chandigarh, India; 2 Department of Biotechnology, Goa University, Taleigao Plateau, Goa, India; 3 Department of Pediatrics, Advanced Pediatrics Centre, Post Graduate Institute of Medical Education and Research, Chandigarh, India; 4 Department of Statistics, Panjab University, Chandigarh, India

**Keywords:** Amyotrophic lateral sclerosis, SOD1, PolyPhen2, QMEAN, Ramachandran plot, PHP-SNP

## Abstract

**Background:**

Amyotrophic lateral sclerosis (ALS) is a progressive neurodegenerative fatal disease that can affect the neurons of brain and spinal cord. ALS genetics has identified various genes to be associated with disease pathology. Oxidative stress induced *bunina* and *lewy* bodies formation can be regulated through the action of SOD1 protein. Hence, in the present study we aim to analyse the structural and functional annotation of various reported SOD1 variants throughout and their putative correlation with the location of mutation and degree of ALS severity by inferring the structural and functional alterations in different SOD1 variants.

**Methods:**

We have retrieved around 69 SNPs of SOD1 gene from *Genecards*. Structural annotation of SOD1 variants were performed using *SWISS Model, I*-*Mutant 2.0, Dynamut, ConSurf*. Similarly, the functional annotation of same variants were done using *SIFT, PHP*-*SNP*, *PolyPhen2, PROVEAN* and *RegulomeDB*. Ramachandran plot was also obtained for six synonymous SNPs to compare the amino acid distribution of wild-type SOD1 (WT SOD1) protein. Frequency analysis, Chi square analysis, ANOVA and multiple regression analysis were performed to compare the structural and functional components among various groups.

**Results and conclusion:**

Results showed the mutations in conserved domain of SOD1 protein are more deleterious and significantly distort the tertiary structure of protein by altering Gibb’s free energy and entropy. Moreover, significant changes in SIFT, PHP-SNP, PolyPhen2, PROVEAN and RegulomeDB scores were also observed in mutations located in conserved domain of SOD1 protein. Multiple regression results were also suggesting the significant alterations in free energy and entropy for conserved domain mutations which were concordant with structural changes of SOD1 protein. Results of the study are suggesting the biological importance of location of mutation(s) which may derive the different disease phenotypes and must be dealt accordingly to provide precise therapy for ALS patients.

## Introduction

*Amyotrophic lateral sclerosis* (ALS, also known as Lou Gehrig’s disease), a term coined by Jean Martin Charcot in 1874, is considered to be one of the most fatal neurodegenerative diseases. It can be characterized by progressive degeneration of both upper and lower motor neurons (amyotrophic) leading to hardening of lateral columns and creeping paralytic condition (lateral sclerosis). For ALS patients, maximum duration of survival is around 3–5 years [1, 2]. As the disease progresses, phosphorylation of neurofilaments stimulates the accumulation of bunina and lewy bodies in pathophysiology of ALS patients consequently induce inflammation in perikarya and proximal axons. Spheroids and ubiquitinylated strands can also stimulate the inflammation in these patients later which can be mediated through triggered response of microglia and astrocytes [3]. Terminal stage in ALS patients can feature failure of cardio-respiratory system after 2-5 years of onset of disease, resulting into death [2].

Three phenotypic variants of ALS have been described which include: pacific (associated with dementia), familial (mostly autosomal dominant, fALS), and sALS (sporadic ALS) [1]. Approximate, 10% of familial ALS cases demonstrate the Mendelian inheritance. Interestingly, 20% of fALS cases have exhibited the *SOD1* mutation (superoxide dismutase 1 contains Cu and Zn in catalytic site). Additionally, mutation in *TARDBP* has also accounted for 5-10% of fALS cases along with the mutations in *FUS* (5%) and *ANG* (1%) [4]. However, sALS has also shown 5% cases with SOD1 mutation. Most of SOD1 mutations showed dominant clinical phenotype in ALS disease [5] except D90A mutation which could exhibit either recessive [6] or dominant [7, 8] phenotype. Mutations in 13 genes and their loci have been identified as the causal genetic factor for typical ALS clinical phenotype which primarily involves SOD1, *TARDBP*, ANG, FUS, and OPTN etc. [4]. Recent genome wide association studies (GWAS) have also suggested and associated seven novel genes with ALS pathology including *C21orf2, TUBA4A, CHCHD10, NEK1, TBK1, MATR3,* and *CCNF* [9]. Intriguingly, hexa-nucleotide expansion (G4C2) of C9ORF72 gene (by mean of *loss* or *gain of function* mutations leading to develop somatic mosaicism) has also been associated with fALS (with 3.2% prevalence rate in Indian ALS) which may stimulate pathology by TDP-43 accumulation, impaired RNA metabolism and defective proteosomal degradation mechanism [10–12]. *C9ORF72* association with fALS is signifying the biological importance of non-coding mutation, though it’s not theme of current study.

The present study aims to examine the changes in functional and structural domains of SOD1 protein for the reported SNPs in various studies. Additionally, we also attempted to investigate significance of mutation location in maintaining the free energy and entropy of protein’s tertiary structure. The impact of synonymous SNPs on protein structure as they do not exert any changes in the tertiary structure has also been explored to demonstrate their biological significance in SOD1 protein structure. Moreover, we also tried to decipher the biological significance of individual scores obtained from various bioinformatics tools to show their impact on structural and functional aspects of SOD1 protein and their implication in ALS severity which can be used to differentiate transmittant ALS phenotypes and can provide the substrate for development of personalized medicine.

### Methodology

We have retrieved 69 clinically associated exonic SNPs of *SOD1* with ALS pathology in various populations from *Genecards* [13] (Accession number for protein from NCBI NP_000445.1, Uniprot ID: P00441) (Additional file 1: Table S2**)**. Structural and functional parameters of SOD1 genetic variations were collected from different web servers (Additional file 1: Table S1). *Consurf* [14] was utilized to identify the position of evolutionary conserved amino acid based on their respective SNPs (Additional file 1: Figure S1). FASTA format has been taken into consideration to annotate SOD1 protein structure and its comparison with different variants along with the severity of ALS pathology. Protein Data Bank (PDB) structure models were generated using Swiss Model [15]. All the structural components of protein including the Cβ, all atoms, salvation and torsion angles were retrieved from *Swiss Model* web server. Collectively these values define the Q mean (Z-score) of protein and describe the degree of naiveness of the protein model based on genetic polymorphism and ultimately signifying the protein stability. The corresponding free energy (Gibb’s free energy) and the entropy with changes in protein sequence were also obtained from *Dynamut* [16] and *I*-*Mutant 2.0 [*17*]* (Additional file 1: Table 1). Functional association of free energy and entropy changes can be correlated with protein stability and molecular flexibility of mutated protein. Functional annotation of studied mutations has also been analysed through algorithms derived from *SIFT* [18], *PROVEAN [*19*]* and *PolyPhen*-*2 [*20*]* to express the effect of non-synonymous SNPs (nsSNP) to define deleterious and/or neutral nature of mutation. Moreover, reliability index (RI) were also retrieved through *PhD*-*SNP* [21] to demonstrate the deleterious effect of nucleotide variation (SNPs) to define the nature of the mutation as neutral or diseased (Tables 2, 3 and 4). Predictive functional annotation of SNPs was also determined by using *RegulomeDB* [22]. Scoring of SNPs through *RegulomeDB* can be represented as transcription factor (TF) binding site or regulatory regions (promoter, operator and enhancer sequences) or DNase hypersensitive region etc. Synonymous mutations were scored using *RegulomeDB* where structural and functional annotations cannot be predicted for such variations (Tables 2, 3 and 4). *String* [23] was used to see the biological interaction of SOD1 with other biomolecules which may collectively regulate the oxidative stress and the processes mediated by them. Moreover, to identify the evolutionary conserved pattern of SOD1, cladogram among various species was analyzed using *MEGAX* [24].

**Table 1:** Tabular representation QMEAN, Cβ, solubility and torsion obtained from SWISS model algorithm and domain location of studied SOD1 variants

Mutation	ConSurf	Nature	QM	Cβ	All atom	Solv	Tor	Mutation	ConSurf	Nature	QM	Cβ	All atom	Solv	Tor
L39V	Con.	NP	2.67	2.71	−0.89	0.38	2.15	**V149G**	Con.	NP	2.46	2.98	−0.85	0	2.02
G38R	Con	PB	1.77	2.45	−0.97	0.33	1.28	**G148D**	Con.	PA	2.53	2.7	−1.02	0.39	2.01
Q23R	Con.	PB	2.56	2.68	−0.96	0.36	2.06	**N140L**	Con.	NP	2.37	2.56	−1.09	0.37	1.88
Q23L	Con.	NP	2.89	2.9	−0.96	0.37	2.36	**V149I**	Con.	NP	2.69	2.82	−0.9	0.42	2.14
N87S	Con.	P	2.69	2.75	−0.96	0.33	2.19	**T55R**	Con.	PB	2.72	2.71	−0.9	0.41	2.2
N87I	Con.	NP	2.5	2.64	−0.87	0.46	1.96	**S135N**	Con.	P	2.55	2.73	−0.87	0.33	2.04
G42S	Con.	P	2.04	2.9	−0.92	0.38	1.45	**S135T**	Con.	P	2.72	2.43	−0.93	0.32	2.29
G42D	Con.	PA	2.13	2.87	−0.97	0.38	1.56	**L85V**	Con.	NP	2.67	2.72	−0.93	0.37	2.16
H44R	Con.	PB	2.36	2.51	−0.93	0.33	1.89	**G17S**	Con.	P	2.52	2.65	−1.04	0.23	2.07
G86S	Con.	P	2.01	2.79	−0.9	0.28	1.48	**G17C**	Con.	P	2.55	2.57	−1.07	0.21	2.13
G86R	Con.	PB	2.35	2.39	−1.06	0.32	1.92	**L127S**	Con.	P	2.9	3.11	−0.8	0.33	2.33
L107V	Con.	NP	2.73	2.8	−0.88	0.35	2.21	**I114T**	Avg.	P	2.75	2.69	−0.92	0.29	2.28
L107F	Con.	NP	2.45	2.28	−0.89	0.33	2.03	**I105F**	Avg.	NP	2.65	2.57	−0.94	0.27	2.21
A5V	Con.	NP	2.55	2.79	−0.92	0.23	2.07	**C7T**	Avg.	P	2.44	2.75	−0.9	0.27	1.95
H47R	Con.	PB	2.72	2.71	−0.91	0.24	2.26	**C7F**	Avg.	NP	2.43	2.41	−0.96	0.1	2.07
A5T	Con.	P	2.56	2.56	−0.97	0.24	2.13	**I152T**	Avg.	P	2.59	2.71	−0.88	0.34	2.08
A5S	Con.	P	2.82	2.65	−0.93	0.32	2.34	**G13R**	Avg.	PB	2.52	3.09	−0.87	0.59	1.84
A146T	Con.	P	2.52	2.84	−1.05	0.26	2.02	**S106L**	Avg.	NP	2.49	2.82	−0.98	0.36	1.96
G73S	Con.	P	2.42	2.46	−0.92	0.26	1.99	**S106L**	Avg.	NP	2.49	2.82	−0.98	0.36	1.96
G73C	Con.	P	2.54	2.45	−0.91	0.3	2.1	**I113T**	Avg.	P	2.7	2.76	−0.97	0.34	2.19
F46S	Con.	P	2.76	2.84	−0.93	0.35	2.24	**I36F**	Avg.	NP	2.64	2.61	−1.08	0.29	2.19
F46C	Con.	P	2.79	2.75	−0.96	0.37	2.29	**F65L**	Avg.	NP	2.71	2.89	−0.89	0.36	2.17
H81R	Con.	PB	2.69	2.79	−0.89	0.3	2.18	**E101G**	Var	NP	2.53	2.49	−1.01	−0.05	2.21
I150T	Con.	P	2.47	2.69	−0.92	0.34	1.97	**L145S**	Var	P	2.61	2.71	−0.86	0.32	2.12
V119L	Con.	NP	2.57	2.71	−0.88	0.35	2.06	**E22K**	Var	PB	2.61	2.47	−1.12	0.37	2.16
A90V	Con.	NP	2.82	2.8	−1.03	0.22	2.36	**D97N**	Var	P	2.67	2.71	−0.87	0.3	2.18
R116G	Con.	NP	2.82	2.59	−0.93	0.41	2.33	**L145F**	Var	NP	2.66	2.85	−0.91	0.36	2.12
R116C	Con.	P	2.56	2.57	−0.98	0.47	2.04	**G74R**	Var	PB	2.49	2.59	−0.98	0.24	2.04
L85F	Con.	NP	2.6	2.71	−0.93	0.28	2.12	**D91A**	Var	NP	2.52	2.99	−0.78	0.27	1.98
V88A	Con.	NP	2.57	2.68	−0.89	0.22	2.11	**N20S**	Var	P	2.7	2.76	−0.81	0.36	2.18
I150T	Con.	P	2.47	2.69	−0.92	0.34	1.97

**Table 2:** Tabular representation of PROVEAN, SIFT and PolyPhen-2 scores along with Gibb’s free energy and entropy values for studied variants which fall in conserve domain of SOD1 protein

Mutation	DDG (Kcal/mol)	Stability	ΔΔG ENCoM (kcal/mol)	Stability	ΔΔSVib ENCoM (kcal.mol^−^1.K^−^1)	Mol flex	Provean	Eff.	RI	Eff.	SIFT	Eff.	Pphen2	Eff.	Reg
L39V	−0.84	Dec.	0.01	D	−0.013	Dec.	−2.77	Del	6	Dis	0.02	APF	0.998	Pro. D	5
G38R	−1.14	Dec.	0.32	D	−0.406	Dec.	−7.17	Del	8	Dis	0	APF	1	Pro. D	5
Q23R	−1.26	Dec.	−0.01	D	0.007	Inc.	−3.18	Del	7	Dis	0	APF	0.018	Ben	4
Q23L	−0.01	Dec.	−0.144	D	0.180	Inc.	−4.76	Del	5	Dis	0.01	APF	0.117	Ben	4
N87S	−0.47	Dec.	−0.05	D	0.066	Inc.	−4.94	Del	6	Dis	0	APF	1	Pro. D	4
N87I	1.44	Inc.	0.19	D	−0.241	Dec.	−8.91	Del	7	Dis	0	APF	1	Pro. D	4
G42S	−0.27	Dec.	−0.03	D	0.032	Inc.	−5.57	Del	7	Dis	0.01	APF	0.999	Pro. D	5
G42D	−1.09	Dec.	0.03	D	−0.038	Dec.	−6.52	Del	6	Dis	0	APF	0.939	Pos. D	5
H44R	−0.86	Dec.	0.17	D	−0.206	Dec.	−7.40	Del	7	Dis	0.26	Tol	1	Pro. D	5
G86S	−2.02	Dec.	0.43	D	−0.541	Dec.	−5.97	Del	8	Dis	0	APF	1	Pro. D	4
G86R	−1.71	Dec.	0.97	S	−1.209	Dec.	−7.91	Del	8	Dis	0.03	APF	1	Pro. D	4
L107V	−1.01	Dec.	−0.40	D	0.499	Inc.	−2.96	Del	6	Dis	0.01	APF	0.997	Pro. D	4
L107F	−0.47	Dec.	0.581	S	−0.726	Dec.	−3.93	Del	8	Dis	0	APF	1	Pro. D	4
A5V	−0.62	Dec.	0.95	S	−1.186	Dec.	−3.20	Del	7	Dis	0	APF	0.999	Pro. D	4
H47R	−0.75	Dec.	0.53	S	−0.663	Dec.	−7.52	Del	7	Dis	0	APF	1	Pro. D	5
A5T	−0.55	Dec.	0.39	D	−0.485	Dec.	−3.08	Del	7	Dis	0	APF	0.993	Pro. D	4
A5S	−0.84	Dec.	0.04	D	−0.045	Dec.	−2.30	Neu	7	Dis	0	APF	0.393	Ben	4
A146T	−0.68	Dec.	0.384	D	−0.48	Dec.	−3.39	Del	7	Dis	0	APF	1	Pro. D	4
G73S	−0.05	Dec.	0.036	D	−0.045	Dec.	−2.80	Del	7	Dis	0	APF	1	Pro. D	4
G73C	0.07	Inc.	−0.14	D	0.18	Inc.	−2.78	Del	5	Dis	0.02	APF	0.999	Pro. D	4
F46S	−2.78	Dec.	−0.08	D	0.096	Inc.	−2.70	Del	7	Dis	0	APF	0.749	Pos. D	4
F46C	−1.44	Dec.	0.26	D	−0.326	Dec.	−5.06	Del	7	Dis	0	APF	0.998	Pro. D	4
H81R	−1.45	Dec.	0.21	D	−0.265	Dec.	−7.33	Del	7	Dis	0	APF	1	Pro. D	4
I150T	−2.84	Dec.	−0.22	D	0.274	Inc.	−5.76	Del	8	Dis	0	APF	1	Pro. D	2b
V119L	−0.43	Dec.	0.28	D	−0.345	Dec.	−5.55	Del	8	Dis	0	APF	0.97	Pro. D	4
A90V	−0.7	Dec.	0.33	D	−0.407	Dec.	−8.41	Del	8	Dis	0	APF	1	Pro. D	4
R116G	−2.02	Dec.	−0.83	D	1.033	Inc.	−7.50	Del	8	Dis	0	APF	0.999	Pro. D	5
R116C	−2.12	Dec.	−0.61	D	0.759	Inc.	−7.51	Del	7	Dis	0	APF	1	Pro. D	5
L85F	−1.03	Dec.	−0.25	D	0.313	Inc.	−7.87	Del	8	Dis	0	APF	1	Pro. D	4
V88A	−3.18	Dec.	−0.12	D	0.154	Inc.	−4.17	Del	8	Dis	0	APF	1	Pro. D	3a
I150T	−1.53	Dec.	0.396	D	−0.495	Dec.	−2.95	Del	5	Dis	0	APF	1	Pro. D	4
V149G	−1.13	Dec.	0.58	S	−0.724	Dec.	−3.88	Del	7	Dis	0.21	Tol	1	Pro. D	3a
G148D	−0.88	Dec.	−0.41	D	0.51	Inc.	−6.92	Del	9	Dis	0	APF	1	Pro. D	4
N140L	−1.13	Dec.	−0.15	D	0.188	Inc.	−7.91	Del	7	Dis	0	APF	1	Pro. D	4
V149I	−0.73	Dec.	0.39	D	−0.482	Dec.	−3.83	Del	6	Dis	0	APF	1	Pro. D	4
T55R	−1.96	Dec.	−0.38	D	0.476	Inc.	−3.96	Del	0	Neu	0	APF	0.999	Pro. D	3a
S135N	−3.18	Dec.	−0.12	D	0.154	Inc.	−4.17	Del	8	Dis	0	APF	1	Pro. D	3a
S135T	−2.62	Dec.	−0.33	D	0.414	Inc.	−6.08	Del	7	Dis	0	APF	1	Pro. D	3a
L85V	−1.11	Dec.	0.043	D	−0.054	Dec.	−5.92	Del	7	Dis	0	APF	1	Pro. D	
G17S	1.67	Inc.	−0.17	D	0.214	Inc.	−8.30	Del	5	Dis	0	APF	1	Pro. D	4
G17C	−0.55	Dec.	−0.05	D	0.064	Inc.	−0.87	Neu	5	Neu	0.43	Tol	1	Pro. D	3a
L127S	0.55	Inc.	0.105	D	−0.132	Dec.	−5.66	Del	5	Dis	0.02	APF	0.869	Pos. D	2b

**Table 3:** Tabular representation of PROVEAN, SIFT and PolyPhen-2 scores along with Gibb’s free energy and entropy values for studied variants which fall in average domain of SOD1 protein

Mutation	DDG (Kcal/mol)	Stability	ΔΔG ENCoM kcal/mol	Stability	ΔΔS_Vib_ ENCoM (kcal.mol^−1^.K^−1)^	Mol flex	Provean	Eff.	RI	Eff.	SIFT	Eff.	Pphen2	Eff.	Reg.
I114T	−1.82	Dec	−0.345	Destab	0.43	Inc	−4.85	Del	4	Dis	0	APF	0.999	Pro. D	2b
I105F	−3.1	Dec	0.395	Destab	−0.49	Dec	−3.61	Del	8	Dis	0	APF	0.999	Pro. D	4
C7T	−0.13	Dec	0.114	Destab	−0.14	Dec	−7.92	Del	4	Dis	0.06	Tol	1	Pro. D	4
C7F	0.7	Inc	1.306	Stab	−1.63	Dec	−8.79	Del	8	Dis	0.01	APF	1	Pro. D	4
I152T	−3.12	Dec	−0.177	Destab	0.221	Inc	−3.98	Del	4	Dis	0.03	APF	0.969	Pro. D	4
G13R	−1.96	Dec	0.171	Destab	−0.21	Dec	−2.99	Del	6	Dis	0.03	APF	0.968	Pro. D	4
S106L	−2.1	Dec	−0.153	Destab	0.19	Inc	−4.89	Del	5	Dis	0.08	Tol	0.924	POS. D	4
S106L	−2.1	Dec	−0.153	Destab	0.19	Inc	−4.89	Del	2	Dis	0.08	Tol	0.924	POS. D	4
I113T	−2.42	Dec	−0.209	Destab	0.26	Inc	−4.72	Del	6	Dis	0	APF	1	Pro. D	2b
I36F	−0.51	Dec	0.517	Stab	−0.65	Dec	−3.46	Del	7	Dis	0.02	APF	0.998	Pro. D	4
F65L	−1.44	Dec	−0.396	Destab	0.495	Inc	−5.70	Del	5	Dis	0	APF	0.995	Pro. D	5

**Table 4:** Tabular representation of PROVEAN, SIFT and PolyPhen-2 scores along with Gibb’s free energy and entropy values for studied variants which fall in variable domain of SOD1 protein

Mutation	DDG (Kcal/mol)	Stability	ΔΔG ENCoM kcal/mol	Stability	ΔΔS_Vib_ ENCoM (kcal.mol^−1^.K^−1)^	Mol flex	Provean	Eff.	RI	Eff.	SIFT	Eff.	Pphen2	Eff.	Reg.
E101G	−1.46	Dec	−0.381	Destab	0.476	Inc	−3.072	Del	2	Dis	0.18	Tol	0.439	Ben	4
L145S	−2.75	Dec	−0.523	Destab	0.654	Inc	−4.146	Del	6	Dis	0.05	Tol	0.999	Pro d	4
E22K	−1.14	Dec	0.084	Destab	−0.104	Dec	−2.154	Neu	2	Neu	0.32	Tol	0.9	POS D	4
D97N	−2.41	Dec	0.108	Destab	−0.135	Dec	−0.31	Neu	5	Neu	0.44	Tol	0	Ben	4
L145F	−1.13	Dec	0.382	Destab	−0.478	Dec	−2.951	Del	8	Dis	0.06	Tol	0.999	Pro d	4
G74R	−0.71	Dec	1.48	Stab	−1.85	Dec	−5.79	Del	4	Dis	0.06	Tol	1	Pro d	
D91A	−2.46	Dec	−0.148	Destab	0.185	Inc	−2.437	Neu	6	Dis	0.13	Tol	0	Ben	3a
N20S	−0.39	Dec	−0.031	Destab	0.038	Inc	−1.081	Neu	9	Neu	0.4	Tol	0.003	Ben	4

### Statistical analysis

Frequencies and their association were calculated using Fisher’s test analysis. One-way ANOVA was employed to examine the changes in the structural and functional parameters including *QMEAN, Gibb’s free energy* (ΔΔG), entropy (ΔS)*, SIFT and PROVEAN* scores. Independent *t* test was also carried out to calculate the mean difference of the above mentioned parameters between the groups classified based on the location of the amino acid (*e.g.* conserved and variable region). Logistic regression analysis was also conducted to identify the changes in various parameters by considering one of them as dependent factor and to demonstrate their diagnostic efficacy in identifying ALS cases more precisely. We calculated fold changes in both structural and functional parameters by deducing the values of mutant SNPs from wild type (WT).

## Results

### Frequency and association of SOD1 variants

Studied exonic mutations are mostly falling in conserved domain (49 variants) (by *ConSurf*) of SOD1 protein which have been found to affect the protein structure (52 variants) (by *SIFT*) and show deleterious effect (62 variants) (by *PROVEAN*) on SOD1 protein in ALS pathology (Table 5). Gibb’s free energy (*EcoDDG*) of these variants has also suggested that 60 variants are destabilizing the protein structure, though entropy changes have shown both decreasing (n = 38) and increasing (n = 30) trends in almost equal proportions. Results of *RegulomeDB* has also indicated that the most of these variants are falling under the score 4 (n = 46) which represents the binding site for TF and DNAse peak, on the contrary to lesser frequencies of 2b (TF binding + matched TF motif + matched DNase Footprint + DNase peak), 3a (TF binding + any motif + DNase peak) (n = 11) and 5 (TF binding or DNase peak) (n = 9).

**Table 5:** Frequency of studied SOD1 variants based on their structural and functional annotation parameters by various bioinformatics tools

Parameter	Component(s)	Frequency
Domain location	Variable	8
	Average	11
	Conserve	49
PROVEAN phenotype	Deleterious	62
	Neutral	6
SIFT phenotype	Affect protein	52
	Tolerant	16
DDG iMutant stability	Decrease	63
	Increase	5
EcoDDG stability	Destabilize	60
	Stabilize	8
Eco entropy dynamut Flexibility	Decrease	38
	Increase	30
HumDiv phenotype	Benign	7
	Possibly damaging	8
	Probably damaging	53
RegulomeDB	2b + 3a	11
	4	46
	5	9

Interestingly, Fisher’s test analysis has revealed that PROVEAN (p = <0.0001), SIFT (p = <0.0001) and PHD-SNP (p = 0.002) significantly associated with the conserved domain of SOD1 protein which may define the severity of ALS phenotype. Additionally, HumDiv phenotype to predict the PolyPhen-2 model has demonstrated that around 41 SOD1 variants are falling in conserved domain of protein and signifying most of SOD1 variants are probably damaging in nature (Table 6).

**Table 6:** Frequency distribution of various structural and functional parameters among groups classified based on mutation location (conserved or variable domains) and their association by Fisher’s exact test

Parameters	Number	Domain location			P
		Variable	Average	Conserve	
Nature of Mutation	Polar	3	6	18	0.806
	Non Polar	3	4	21	
	Charged	2	1	10	
PROVEAN Phenotype	Deleterious	4	11	47	<0.0001
	Neutral	4	0	2	
PHD-SNP Phenotype	Disease	5	11	47	0.002
	Neutral	3	0	2	
SIFT Phenotype	Affect Protein	0	8	44	<0.0001
	Tolerant	8	3	5	
DDG iMutant Stability	Decrease	8	10	45	0.694
	Increase	0	1	4	
EcoDDG Stability	Destabilize	7	9	44	0.757
	Stabilize	1	2	5	
EcoEntropy Dynamut Flexibility	Decrease	4	5	29	0.666
	Increase	4	6	20	
HumDiv Phenotype	Benign	4	0	3	0.002
	Possibly Damaging	1	2	5	
	Probably Damaging	3	9	41	
RegulomeDB	2b + 3a	1	2	8	0.769
	4	6	8	32	
	5	0	1	8	

Phylogenetic relationship through Cladogram has revealed that human SOD1 is closely related to Pongo abelli, Similarly SOD1 of Rattus norvegicus has also showed phyologenetic proximity to Mus musculus, however, both showed some divergence from the human SOD1 (Additional file 1: Figure S2).

### Alterations of structural and functional parameters

#### Mutation in conserved domain influences PROVEAN score

**Fig. 1:**
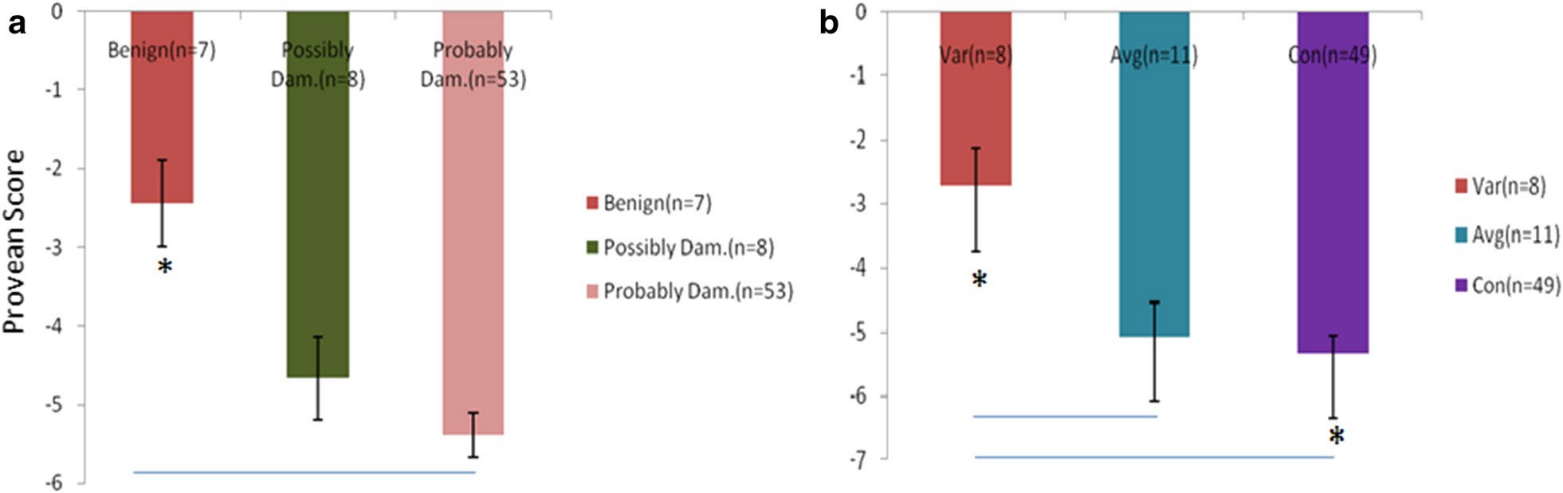
PROVEAN score alterations among **a** HumDiv phenotypes and **b** conserved domain based groups. Var: variable domain (n = 8); Avg: average domain (n = 11) analysed by ANOVA; Con: conserved domain (n = 49). Bar is representing SEM; P value * < 0.05, ** < 0.001, *** < 0.0001

Comparative analysis of structural and functional values derived from various annotating software like Dynamut, PolyPhen-2, Reliability index (RI) of PHD-SNP, PROVEAN and SIFT have indicated significant alterations based on the location and nature of mutations (both in nucleotide and amino acid) in SOD1 protein. PROVEAN score has been found to be significantly varied in HumDiv phenotypes *i.e.* benign, possibly damaging and probably damaging (Fig. 1a) and conserved domains (Fig. 1b). Significant alteration in PROVEAN score has been found (between probably damaging and benign) among HumDiv phenotypes derived from PolyPhen-2 algorithm in SOD1 variants. Similarly, PROVEAN score has also significantly changed in both average and conservative domains as compared to benign domain. Results suggest that biological significance of mutations located in conserved domain which could lead to drastic changes in structural and functional components of SOD1 protein can be correlated with the phenotypic severity of ALS patients.

#### SOD1 Mutation in conserved domain enhances ALS severity by disrupting structural and functional parameters of protein

**Fig. 2:**
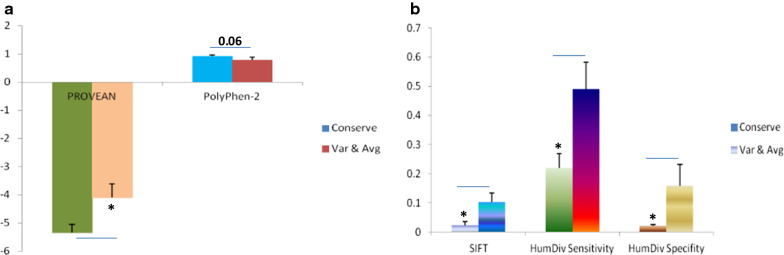
Structural and functional parameters comparison of SOD1 variants fall in conserved and variable-average domains analyzed by independent T-test. **a** PROVEAN and PolyPhen-2, **b** Comparison of SIFT, HumDiv sensitivity and HumDiv specificity in between conserved and variable-average domains. Var-avg: variable-average domains (n = 19); Conserved domain (n = 49); Bar is representing SEM; P value * < 0.05, ** < 0.001, *** < 0.0001

To compare the effect of SOD1 mutant variants in conserved domain versus variable-average region of protein, ANOVA results have significantly changed in PROVEAN and SIFT scores. Functional annotation of SOD1 mutations through PROVEAN and SIFT algorithms are suggesting that changes in structural and functional parameters of SOD1 protein can be correlated with the ALS severity and distorted version of protein which can be corresponding to the location of mutation (conserved versus variable-average) (Fig. 2). Marginally significant alteration reliability index (RI) of PolyPhen-2 has also been observed between conserved versus variable-average domain. Significant alterations in HumDiv sensitivity and specificity derived from PolyPhen-2 algorithm has also suggested highly distorted structure of SOD1 protein when mutation was located in conserved domain as compared to variable-average (Fig. 2).

#### SOD1 variants alter structural component Cβ

**Fig. 3:**
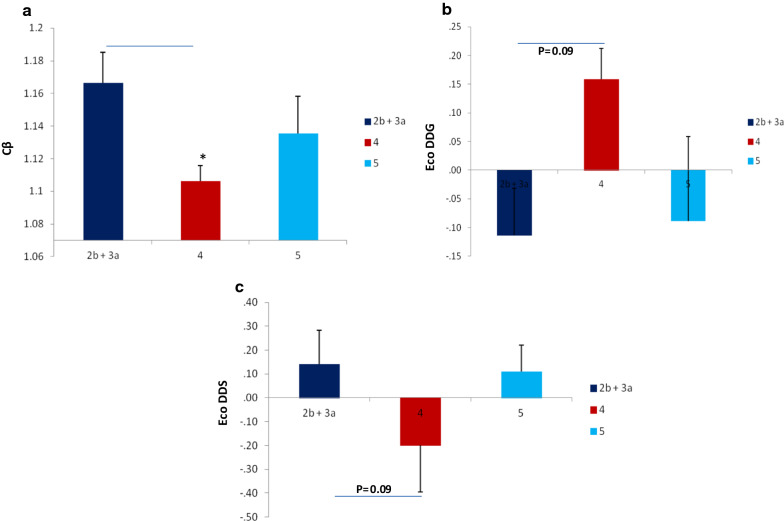
SOD1 variants fall in conserved domain can alter the action of regulatory sequences by distorting the protein structure. **a** Significant changes in Cβ between 2b + 3a and 4 RegulomeDB score. (**b, c**) Gibb’s free energy and entropy alterations among RegulomeBD groups. p-value computed by employing ANOVA. Bar is representing SEM; P value * < 0.05, ** < 0.001, *** < 0.0001. RegulomeDB 2b + 3a (n = 11); RegulomeDB 4 (n = 46); RegulomeDB 5 (n = 9)

Results of *Pearson’s chi*- *square* have shown that most of the SOD1 variants are located in conserved domain of protein. ANOVA results have demonstrated that the structural component *of* SOD1 protein, namely *Cβ,* derived from SWISS model has significantly altered in *RegulomeDB* 4 score as compare to score 2b +3a (Fig. 3a). Alterations in ΔΔG ENCoM (kcal/mol) and ΔΔS_Vib_ ENCoM (kcal.mol^−1^.K^−1^) have also been observed among RegulomeDB groups, though not of statistical significance (Fig. 3b, c). Results are indicating that the conserved domain mutation(s) can alter the structural component *Cβ* of SOD1 protein and may influence the binding of TF and DNase hypersensitivity.

The impact of various algorithms to predict the functional and structural components was further correlated with SOD1 variants located in different domains including variable and conserved etc. Multiple regression analysis has shown that SWISS model component QMEAN has been found to be associated with PROVEAN score and can alter the same by 3.299 (p = 0.003). Moreover, ΔΔG ENCoM (kcal/mol) has also exhibited the association with QMEAN (p = 0.016; B = −0.156). Importantly, standardized coefficient *beta (β)* has also demonstrated the difference of 0.358. Entropy changes (ΔΔS_Vib_ ENCoM (kcal.mol^−1^K^−1^) of SOD1 variants showed the significant association with DDG value derived from *iMutant* (p = 0.02) and can alter the value by −0.582 unit. Results are suggesting the alterations in corresponding stability and flexibility of SOD1 protein with respect to nature and location of mutation. Importantly, predicted structural and functional changes of SOD1 protein can be further correlated with expression levels in ALS pathology which may be useful for predicting precise ALS phenotype (Table 7).

**Table 7:** Multiple regression analysis to demonstrate the association of structural and functional parameters of SOD1 variants and their correlation with ALS

Coefficients					
Dependent variable: PROVEAN Score					
	Unstandardized coefficients		Standardized coefficients	T	P
	B	Std. Error	beta		
(Constant)	−13.98	2.891		−4.836	0
QMEAN	3.299	1.058	0.358	3.119	*0.003*
Dependent variable: QMEAN					
(Constant)	2.921	0.065		44.757	0
PROVEAN Score	0.037	0.012	0.336	3.025	*0.004*
Eco DDG DynaMut	−0.156	0.063	−0.274	−2.47	*0.016*
Dependent variable: DDG iMutant value					
(Constant)	−1.376	0.127		−10.811	0
Eco Entropy Dynamut	−0.582	0.244	−0.281	−2.382	*0.02*
Dependent variable: PolyPhen-2 Score					
(Constant)	0.621	0.076		8.14	0
PROVEAN Score	−0.054	0.014	−0.429	−3.863	*0.02*

Structural and functional annotations of synonymous SNPs (sSNPs) cannot be done with existing tools to predict their structural and functional alterations. In current data set, six synonymous SOD1 variants were retrieved which were located in TF binding site and DNase hypersensitivity (Regulome 4 score). Interestingly, five out of six SOD1 sSNPs were falling in conserved domain and one was located in variable region. Results are suggesting the regulatory function of sSNPs by modulating the SOD1 expression and associated cellular mechanism (Table 8).

**Table 8:** Clinically associated synonymous SNPs of SOD1 in ALS pathology with their Consurf and RegulomeDB score

Mutation	Codon	ConSurf	Regulome score
L85L	253T > C	Conserve	4
L85L	255G > A	Conserve	4
A141A	423T > A	Conserve	4
N140N	420C > T	Conserve	4
E22E	66G > A	Variable	4
N132N	396T > C	Conserve	4

Moreover, it was found that SNP at 94 position of SOD1 protein has six different variants as reported by various studied. (Table 9), suggesting a potential hot spot in ALS pathology. Comparative analysis has indicated that structural and functional annotations of these variants have drastically changed as compared to WT protein structure of SOD1. Results have shown increased scores of QMEAN, Cβ, salvation and torsion angel parameters as compared to WT SOD1 protein. All structural factors derived from SWISS modeling in WT protein structure are required to maintain amino acids geometry, for necessary hydrogen and hydrophobic bonding to guide the secondary and tertiary structures of SOD1 protein. Predictive ΔΔG ENCoM (kcal/mol) and ΔΔSVib ENCoM (kcal.mol^−^1.K^−^1) by *Dynamut* also got altered which was concordant with structural changes in protein derived from these SOD1 variants. Results can indicate the distorted molecular flexibility and decreased stability of SOD1 protein derived from these six SNPs. Importantly, these variants were falling in the conserved region of SOD1 protein and primarily affect the TF binding and DNase hypersensitivity.

**Table 9:** Tabular representation of PROVEAN, SIFT and PolyPhen-2 scores along with Gibb’s free energy, entropy values and Ramachandran plot details for studied variants which fall at 94 position of SOD1 protein

Mutation	ΔΔG ENCoM (kcal/mol)	Stability	ΔΔSVib ENCoM (kcal.mol^−^1.K^−^1)	Mol flex	Provean	RI	Eff.	SIFT	Eff	Pphen2	Eff.	Reg.	QM	Cβ	All Atoms	Solv	Tor
Wild Type													0.93	2.41	−1.29	−0.13	0.58
G94S	0.27	D	−0.335	Dec.	−5.33	7	Dis	0.05	Tol	0.856	Pos. D	4	2.17	2.68	−0.93	0.28	1.67
G94R	0.25	D	−0.315	Dec.	−7.22	8	Dis	0	APF	1	Pro. D	4	2.21	2.69	−0.96	0.4	1.67
G94C	0.20	D	−0.245	Dec.	−8.18	9	Dis	0.05	Tol	1	Pro. D	4	2.32	2.8	−0.91	0.31	1.8
G94N	0.18	D	−0.222	Dec.	−5.16	8	Dis	0	APF	0.664	Pos. D	4	2.32	2.76	−0.93	0.35	1.79
G94A	−0.05	D	0.067	Inc.	−5.48	6	Dis	0.04	APF	0.991	Pro. D	4	2.28	2.53	−0.51	−0	1.91
G94V	0.38	D	−0.471	Dec.	−8.27	8	Dis	0	APF	1	Pro. D	4	2.08	2.68	−0.96	0.35	1.56

Mutation	ConSurf	Nature	No. of res. in favored Reg	No. of res. in allowed Reg	No. of res. in outlier Reg
Wild Type	Con	P	286	14	2
G94S	Con.	P	301	3	0
G94R	Con.	PB	301	3	0
G94C	Con.	P	301	3	0
G94N	Con.	P	301	3	0
G94A	Con.	NP	299	3	0
G94V	Con.	NP	303	1	0

**Fig. 4:**
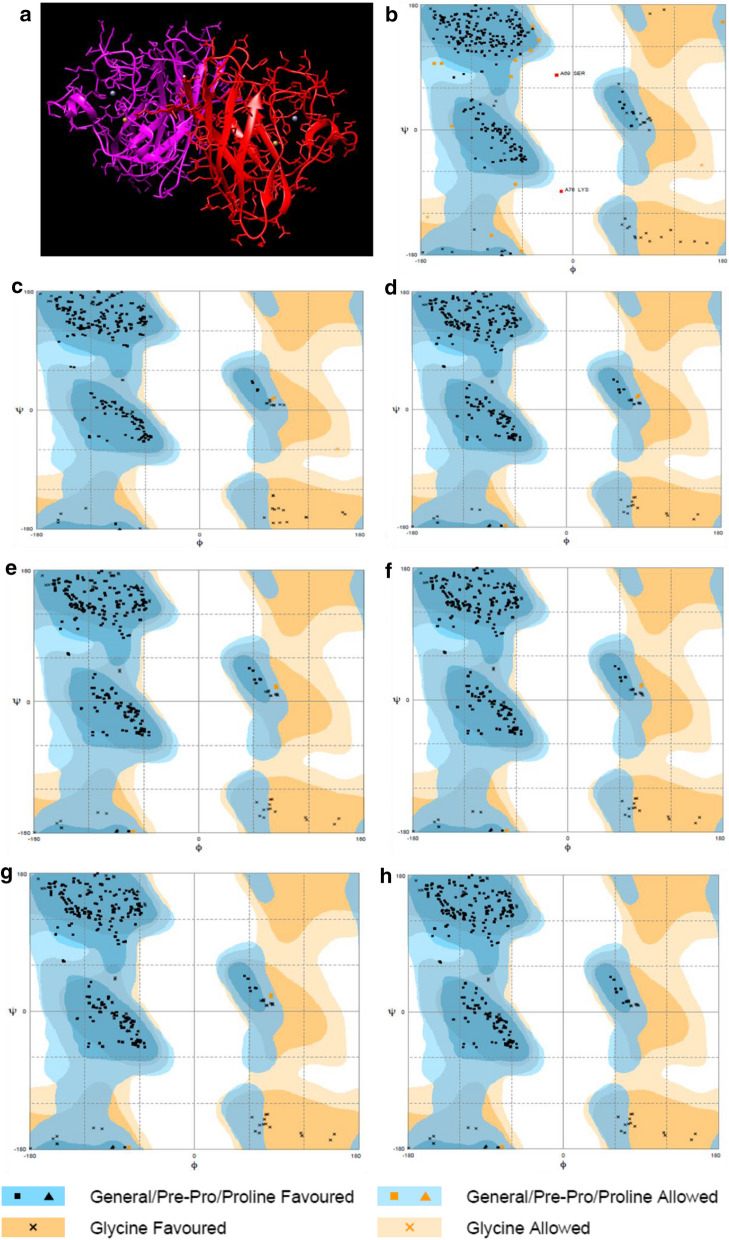
Ramachandran plot to demonstrate the distribution of Gly, Pre-Pro, and Pro in allowed and favoured regions for multiple variants at 94 position require to maintain the secondary structure SOD1 protein

Comparative Ramachandran plot analysis has also suggested the changes in distribution of amino acids in favored, allowed and outlier regions as shown in Fig. 4. Results of Table 9 demonstrating the increased number of amino acids fall in favored region in these six SNPs as compared to WT (286 amino acids). Similarly, drastic changes in number of amino acids can be seen in SOD1 variants in both allowed and outlier regions of these six SNPs in comparison to WT (14 and 2, respectively). Results signify changes in structural parameters in all six variants as compared to WT which can affect the bonding pattern of secondary structure SOD1 proteins.

## Discussion

**Fig. 5:**
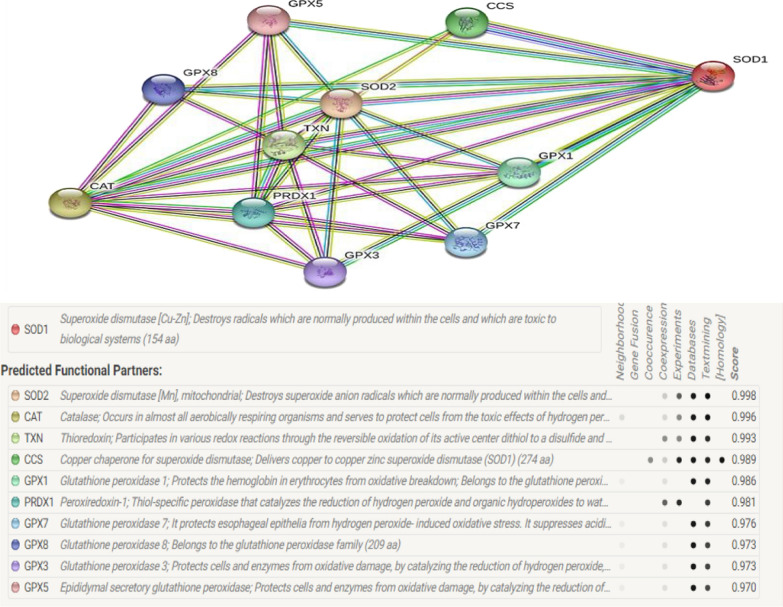
Schematic illustration of various biomolecules to reveal the interaction with SOD1 protein to perform the various cellular and molecular function to maintain oxidative homeostatis

Present study provides the comparative analysis of various bioinformatics tools used to predict structural and functional aspects of exonic variants of SOD1 in ALS pathology which may be used to decipher the clinical severity of disease and translational implications. Not much success has been achieved in the advancement of prognostic and diagnostic fields to predict ALS in early stages of pathology. ALS is one of the most devastating degenerative diseases which demands faster conversion of genetic data into its clinical and translational development. ALS patients have been prescribed Riluzole to offer symptomatic relief and retard the degenerative process by inhibiting release of glutamic acid and noncompetitive action with N-methyl-D-aspartate (NMDA) receptors [25, 26]. We have demonstrated that mutation(s) found in conserved domain of SOD1 protein are more deleterious and disease causing which can significantly distort the structure of SOD1 protein by altering Gibb’s free energy and entropy of naïve protein. PROVEAN, PHD-SNP and SIFT scores also got altered significantly in mutations located in the conserved domain of SOD1 protein. Interestingly, results of multiple regression analysis to see individual impact of different entities including SIFT, PROVEAN, Polyphen2, QMEAN etc. on structure and function of SOD1 protein have revealed significant changes in free energy and entropy (Delta G and Delta S) which were concordant with structural changes in SOD1 protein. Results are suggesting that conserved domain mutation may have pivotal role in balancing the free energy and entropy of SOD1 protein by maintaining homeostatic interactions. Multiple bioinformatics tools to predict the structural and functional analysis can enhance the possibility of identifying variant’s nature that can be missed by employing one tool to be specific. The resulted SNPs can induce the unfavorable conformational changes in SOD1 protein and may refuse to interact with other associated molecules which may hamper the mediated functions of downstream molecules (Fig. 5). Mutations in conserved domain of SOD1 protein have been found to stimulate the sedimentable aggregates [27], impair the activity of Na^+^/K^+^ATPase-α3 [28], reduce the affinity for Zn ion [29] and increase the Palmitoylation [30]. Therefore, it can also be argued that location and degree of mutation of SOD1 gene may have diverse impact on structural and functional aspects of SOD1 protein. This suggests that ALS pathology derived through various mutation may be dealt accordingly to the nature of SOD1 mutation and therapeutic regimen must be designed accordingly. It is evident from our results that mutation in phylogenetically conserved region is pronounced to be highly detrimental in nature because these alterations are located in functional domain of the effective protein and suggest maximum structural distortion of protein. Based on these results, it may be suggestive to provide the therapies or molecules which may assist in maximum structural restoration of the mutated SOD1 protein to provide the interactive interface for downstream molecules, may be beneficial for ALS patients.

Study has also provided comparative analysis of synonymous SNPs (six), though they do not exert any changes in tertiary structure of protein. Our results are suggesting the changes in QMEAN, Cβ, salvation and torsion angle of these six variants as compared to WT SOD1 protein and indicating to consider the same while making a clinical impression of ALS phenotype.

Moreover, Ramachandran plot analysis has also showed the differential distribution of amino acids among favorable, allowed and outlier region require to maintain secondary structure of SOD1 protein suggesting the distorted molecular flexibility and stability of protein of variants at 94 position Such multiple variations at single location may lead to differential clinical phenotypes of ALS based on distribution of amino acid in Ramachandran plot due to varied degree of interactions with downstream molecules.

## Conclusion

Study has indicated the biological significance of mutation fall on conserved domain mutation of SOD1 and can distort the structure of naïve protein. Such spectrum of mutation can confer the various intermittent phenotypes of ALS by exerting the varying degree of interaction with downstream molecules which may warrant the personalized therapy based on location of SOD1 mutation.

## Limitation

Predictive genetic interactions between these SOD1 variants and molecular interaction with other genes have not been deciphered. Corresponding protein levels of SOD1 variants can precisely define consequence in ALS severity and can derive better representation of ALS phenotype which could be demonstrated by adopting cell culture or animal model based analysis.

## Supplementary information

**Additional file 1:** Tables and Figures.

## Data Availability

Data is available with corresponding and first author of the manuscript can be produced whenever required. The accession number for SOD1 protein is NCBI NP_000445.1, Uniprot ID: P00441 (Additional file 1: Table S2). Accession number and other pertaining details from *Genecards* can be seen at link https://www.genecards.org/cgi-bin/carddisp.pl?gene=SOD1#proteins. Retrieval of studied exonic SOD1 variants can be accessed from web portal link https://www.genecards.org/cgi-bin/carddisp.pl?gene=SOD1#snp. Reference sequence of wild type SOD1 was salvaged from NCBI with mentioned link as https://www.ncbi.nlm.nih.gov/protein/NP_000445.1?report=GenPept. Analysis of SOD1 variants were performed from openly available tools and databases which have been cited appropriately in methodology section of the manuscript (Additional file 1: Table S1).

## Abbreviation

ALS: Amyotrophic lateral sclerosis—Motor neuron disease
fALS: Familial ALS—Inherited form of disease
sALS: Sporadic ALS—Non-inherited form of disease, de novo mutation is main cause
SNP: Single nucleotide polymorphism—most common type of genetic variation and being explored in population studies to identified biomarkers
sSNPs: Synonymous SNPs—No change in amino acid after mutation
nsSNP: Non-synonymous SNPs—lead to change in amino acid’s chemical and physical nature after mutation
WT: Wild type—Phenotype occurs in population with maximum frequency
SOD1: Super oxide dismutase 1—Antioxidant enzyme protecting the cell from reactive oxygen species toxicity
TARDBP: TAR DNA binding protein—It acts as neuronal activity response factor in the dendrites of hippocampal neurons
ANG: Angiogenin—Regulate the angiogenesis process
OPTN: Optineurin—Function in cellular morphogenesis and membrane trafficking, vesicle trafficking
C21orf2: Chromosome 21 open reading frame 2—Regulation of cell morphology and cytoskeletal organization
TUBA4A: Tubulin α4a —Major protein of microtubules
CHCHD10: Coiled-coil-helix-coiled-coil-helix domain-containing protein 10—Imperative role in oxidative phosphorylation
NEK1: NIMA Related Kinase 1 (NIMA:never in mitosis gene A)—Regulate neuronal morphology and axonal polarity
TBK1: TANK-binding kinase 1—Serine — threonine protein kinase and regulate cell-proliferation, autophagys and apoptosis. It also involve in anti-viral innate immunity response
MATR3: Matrin-3—It interacts with TDP-43 and provides the structural support to nuclear membrane and shows binding with nucleic acid
CCNF: Cyclin F—Regulate cell cycle transitions through their ability to bind and activate cyclin-dependent protein kinases
GWAS: Genome wide association studies—To explore the genetic variant(s) across the population and their association with the trait in different individuals
ANOVA: Analysis of variance—To analyse the significant differences between the means of two or more independent groups
RI: Reliability index—Predictor of human Deleterious effect of Single Nucleotide Polymorphisms
RC: Ramachandran plot—Analysis of secondary structure of protein by understanding the distribution of *phi-psi* angles
SIFT: The scale-invariant feature transform—To scale the effect of amino acid substitution on protein function
PROVEAN: Protein Variation Effect Analyzer—Amino acid substitution impact on the biological function of a protein
Reg: RegulomeDB Score—Interpretation of regulatory variants in the human genome
ΔΔG: Gibb’s free energy—Equilibrium constant for a reversible reaction
ΔS: Entropy—Degree of randomness
Con.: Conserved—Location of amino acid in conserved domain of SOD1 and consensus present throughout the species
Avg.: Average—Location of amino acid in Average domain of SOD1
Var.: Variable—Location of amino acid in variable domain of SOD1 and can vary throughout the species
P: Polar—Amino acid whose side chain show bonding with water (hydrophilic nature)
NP: Non Polar—Side chain of amino acid buried inside the protein structure and formed hydrophobic bonding
PB: Polar Basic—Polar Amino acid with –NH2 group in the side chain
PA: Polar Acidic—Polar Amino acid with –COOH group in the side chain
Tor: Torsion angle—Dihedral angles in the back bone of protein (*φ*, *ψ*, *ω*)
Solv: Solvation—Bonding between solute and solvent
Mol flex: Molecule flexibility—Molecular flexibility of prot signi the conforma changes to copeup with external sources to distort the protein structure
Eff.: Effect—Effect and indicating the outcome of the mutation
