# Supplementary material for: Does conserved domain SOD1 mutation has any role in ALS severity and therapeutic outcome?
Source: BMC Neurosci. 2020 Oct 9;21:42. doi: 10.1186/s12868-020-00591-3 (PMC7547430; doi:10.1186/s12868-020-00591-3)
Supplement: Supplementary file 1 — Additional file 1: Tables and Figures. [file 12868_2020_591_MOESM1_ESM.docx]

**Supplementary file**

| Analysis | Name | Parameters collected | Web portal | Analysis | Name | Parameters collected | Web portal |
| --- | --- | --- | --- | --- | --- | --- | --- |
| SNP mining | *Gene Cards* | Retrieval of clinically validated exonic SNPs of SOD1 studied in ALS | <https://www.genecards.org/> | **Functional** | *SIFT* | Functional annotation of nonsynonymous mutated protein. | <https://sift.bii.a-star.edu.sg/> |
| Structural | *SWISS Modeling* | Generate protein models in PDB format to visualized protein’s secondary structure | <https://swissmodel.expasy.org/> |  | *PhD-SNP* | To predict the deleterious effect (RI) of nucleotide polymorphism and describes polymorphism as neutral or diseased. | <http://snps.biofold.org/phd-snp/phd-snp.html> |
|  | *I-Mutant 2.0* | Gibb’s free energy with changes in protein sequence (DDG value), stability of protein structure. | <http://folding.biofold.org/i-mutant/i-mutant2.0.html> |  | *PROVEAN* | PROVEAN score describes phenotypic impact of SNPs as neutral or deleterious. | <http://provean.jcvi.org/about.php> |
|  | *Dynamut* | Predict the protein structure stability and flexibility with mutation and provides DDG and DDS values | <http://biosig.unimelb.edu.au/dynamut/> |  | *Polyphen* 2 | Provide the specificity and sensitivity of mutation. | <http://genetics.bwh.harvard.edu/pph2/> |
|  | *ConSurf* | To locate the evolutionary conserve amino acid in protein and nucleotide sequence. | <http://consurf.tau.ac.il/> |  | *RegulomeDB* | Annotate SNPs to predict the functional regulatory region like binding of TF, DNAase hypersensitivity, and promoter regions *etc* | <http://regulome.stanford.edu/index> |

**Table S1:** Tabular representation of bioinformatics tools utilized to annotate functional and structural components of SOD1 variants.

| SNP No. | WT Codon | Mut. Codon | Position | AA(WT) | AA(Mut) | AA Pos. | Type of mutation |
| --- | --- | --- | --- | --- | --- | --- | --- |
| rs121912431 | GGA | AGA | 112G>A | Gly | arg | 38 | Missense |
| rs121912431 | GGA | CGA | 112G>C | Gly | arg | 38 | Missense |
| rs121912432 | CTG | GTG | 115C>G | Leu | val | 39 | Missense |
| rs1169198442 | CAG | CGG | 68A>G | Gln | arg | 23 | Missense |
| rs1169198442 | CAG | CTG | 68A>T | Gln | leu | 23 | Missense |
| rs11556620 | AAT | AGT | 260A>G | Asn | ser | 87 | Missense |
| rs11556620 | AAT | ATT | 260A>T | Asn | ile | 87 | Missense |
| rs121912433 | GGC | AGC | 124G>A | Gly | ser | 42 | Missense |
| rs121912434 | GGC | GAC | 125G>A | Gly | asp | 42 | Missense |
| rs121912435 | CAT | CGT | 131A>G | his | arg | 44 | Missense |
| rs121912436 | GGC | AGC | 256G>A | Gly | ser | 86 | Missense |
| rs121912436 | GGC | CGC | 256G>C | Gly | Arg | 86 | Missense |
| rs121912437 | GGT | AGT | 280G>A | Gly | ser | 94 | Missense |
| rs121912437 | GGT | CGT | 280G>C | Gly | Arg | 94 | Missense |
| rs121912437 | GGT | TGT | 280G>T | Gly | Cys | 94 | Missense |
| rs121912438 | GGT | GAT | 281G>A | Gly | Asp | 94 | Missense |
| rs121912438 | GGT | GCT | 281G>C | Gly | Ala | 94 | Missense |
| rs121912438 | GGT | GTT | 281G>T | Gly | Val | 94 | Missense |
| rs121912439 | GAA | GGA | 302A>G | Glu | Gly | 101 | Missense |
| rs121912440 | CTC | GTC | 319C>G | Leu | Val | 107 | Missense |
| rs121912440 | CTC | TTC | 319C>T | Leu | phe | 107 | Missense |
| rs121912441 | ATT | ACT | 341T>C | Ile | Thr | 114 | Missense |
| rs121912442 | GCC | GTC | 14C>T | Ala | Val | 5 | Missense |
| rs121912443 | CAT | CGT | 140A>G | His | Arg | 47 | Missense |
| rs121912444 | GCC | ACC | 13G>A | Ala | Thr | 5 | Missense |
| rs121912444 | GCC | TCC | 13G>T | Ala | Ser | 5 | Missense |
| rs121912445 | ATC | TTC | 313A>T | Ile | Phe | 105 | Missense |
| rs121912446 | TTG | TCG | 434T>C | Leu | Ser | 145 | Missense |
| rs121912447 | GCT | ACT | 436G>A | Ala | Thr | 146 | Missense |
| rs121912448 | TGC | TAC | 20G>A | Cys | Tyr | 7 | Missense |
| rs121912448 | TGC | TTC | 20G>T | Cys | Phe | 7 | Missense |
| rs121912449 | ATC | ACC | 455T>C | Ile | Thr | 152 | Missense |
| rs121912450 | GAG | AAG | 64G>A | Glu | Lys | 22 | Missense |
| rs121912451 | AGT | AAT | 404G>A | Ser | Asn | 135 | Missense |
| rs121912451 | AGT | ACT | 404G>C | Ser | Thr | 135 | Missense |
| rs121912452 | TTG | CTG | 253T>C | Leu | Leu | 85 | SN Var. |
| rs121912452 | TTG | GTG | 253T>G | Leu | Val | 85 | Missense |
| rs121912453 | GGC | AGC | 49G>A | Gly | Ser | 17 | Missense |
| rs121912453 | GGC | TGC | 49G>T | Gly | Cys | 17 | Missense |
| rs121912454 | TTG | TAG | 380T>A | Leu | *Ter | 127 | Stop GOF |
| rs121912454 | TTG | TCG | 380T>C | Leu | Ser | 127 | Missense |
| rs121912455 | GGT | AGT | 217G>A | Gly | Ser | 73 | Missense |
| rs121912455 | GGT | TGT | 217G>T | Gly | Cys | 73 | Missense |
| rs121912456 | GGC | CGC | 37G>C | Gly | Arg | 13 | Missense |
| rs121912457 | TTC | TCC | 137T>C | Phe | Ser | 46 | Missense |
| rs121912457 | TTC | TGC | 137T>G | Phe | Cys | 46 | Missense |
| rs121912458 | CAT | CGT | 242A>G | His | Arg | 81 | Missense |
| rs121912459 | GAT | AAT | 289G>A | Asp | Asn | 97 | Missense |
| rs1424014997 | ATT | ACT | 449T>C | Ile | Thr | 150 | Missense |
| rs1378590183 | TCA | TTA | 317C>T | Ser | Leu | 106 | Missense |
| rs1235629842 | GTG | CTG | 355G>C | Val | Leu | 119 | Missense |
| rs1235629842 | GTG | TTG | 355G>T | Val | Leu | 119 | Missense |
| rs1280042397 | GCT | GTT | 269C>T | Ala | Val | 90 | Missense |
| rs1301635320 | CGC | GGC | 346C>G | Arg | Gly | 116 | Missense |
| rs1301635320 | CGC | TGC | 346C>T | Arg | Cys | 116 | Missense |
| rs1315541036 | TTG | TTA | 255G>A | Leu | Leu | 85 | SN Var. |
| rs1315541036 | TTG | TTC | 255G>C | Leu | Phe | 85 | Missense |
| rs1339283341 | GTG | GCG | 263T>C | Val | Ala | 88 | Missense |
| rs1378590183 | TCA | TTA | 317C>T | Ser | Leu | 106 | Missense |
| rs1424014997 | ATT | ACT | 449T>C | Ile | Thr | 150 | Missense |
| rs143100660 | GCT | GCA | 423T>A | Ala | Ala | 141 | SN Var. |
| rs1476760624 | GTA | GGA | 446T>G | Val | Gly | 149 | Missense |
| rs1482760341 | TTG | TTC | 435G>C | Leu | Phe | 145 | Missense |
| rs1482760341 | TTG | TTT | 435G>T | Leu | Phe | 145 | Missense |
| rs1555836720 | GGG | AGG | 220G>A | Gly | Arg | 74 | Missense |
| rs1555836950 | GGT | GAT | 443G>A | Gly | Asp | 148 | Missense |
| rs1804449 | AAC | AAA | 420C>A | Asn | Lys | 140 | Missense |
| rs1804449 | AAC | AAT | 420C>T | Asn | Asn | 140 | SN Var. |
| rs567511139 | GTA | ATA | 445G>A | Val | Ile | 149 | Missense |
| rs74315452 | ATC | ACC | 338T>C | Ile | Thr | 113 | Missense |
| rs756458346 | GAG | GGA | 66G>A | Glu | Glu | 22 | SN Var. |
| rs80265967 | GAC | GCC | 272A>C | Asp | Ala | 91 | Missense |
| rs80265967 | GAC | GTC | 272A>T | Asp | Ala | 91 | Missense |
| rs986277034 | ACA | AGA | 164C>G | Thr | Arg | 55 | Missense |
| rs768029813 | AAT | AGT | 59A>G | Asn | Ser | 20 | Missense |
| rs1057524474 | AAT | TTT | 106 A>T | Ile | Phe | 36 | Missense |
| rs1027128618 | AAT | AAC | 396T>C | Asn | Asn | 132 | SN Var. |
| rs1030039318 | TTT | CTT | 193T>C | Phe | Leu | 65 | Missense |

**Table S2:** Details of position and nature of mutation involved in this study retrieved from Genecards.


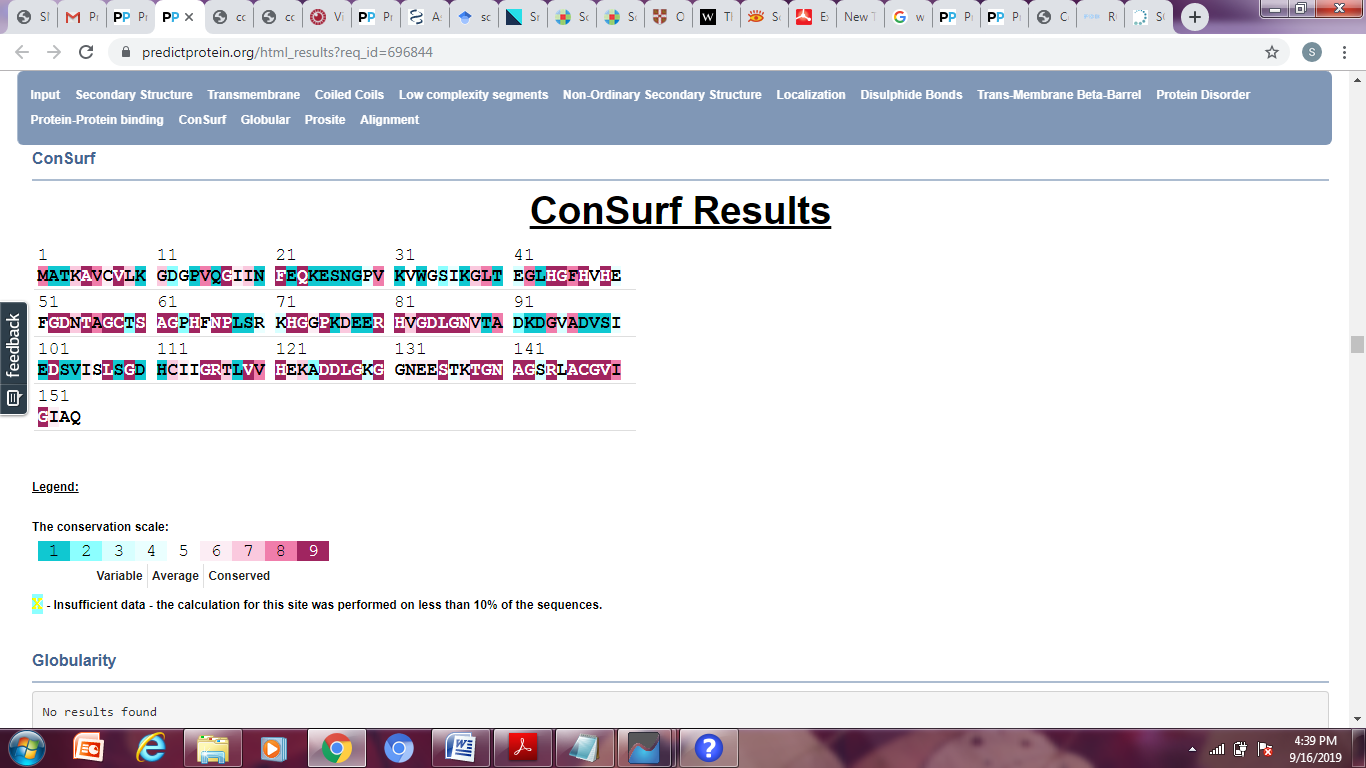


**Figure S1:** Representation of amino acid location on stretch of SOD1 protein sequence derived from ConSurf.


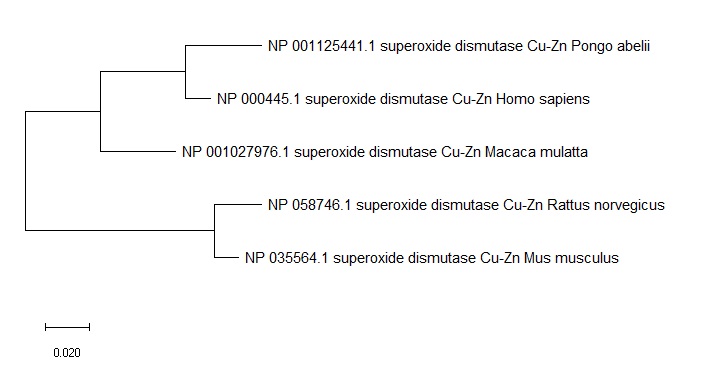


**FigureS2:** Cladogram to demonstrate relationship of SOD1 conserve pattern among higher mammals.
